# Nutritional assessment, multi-platform phytochemical profiling, and biological activities of *Centaurea calcitrapa* L. based on GC–MS and HPLC analysis

**DOI:** 10.1038/s41598-026-60984-y

**Published:** 2026-07-10

**Authors:** Fatma N. Mahmoud, Sahar K. M. Kenawy, Ali A. Badawy, Nashaat N. Mahmoud

**Affiliations:** 1 Botany and Microbiology Department, Faculty of Science (Girls Branch), Al- Azhar University, Cairo, 11754 Egypt; 2 Botany and Microbiology Department, Faculty of Science, Al-Azhar University, Cairo, 11884 Egypt

**Keywords:** *Centaurea calcitrapa* L., Nutritional composition, Phytochemicals, Antioxidant, Antibacterial, Antidiabetic activity, Biochemistry, Biotechnology, Chemistry, Plant sciences

## Abstract

The rise of antibiotic resistance and metabolic disorders has led to increased interest in plant-derived bioactive compounds *Centaurea calcitrapa* L., a medicinal plant rich in phenols and flavonoids, has been traditionally used in folk medicine; however, data regarding the nutritional composition and biological activities of its aerial flowering parts remain limited. This study evaluated the phytochemical profile, nutritional composition, and biological properties of the aerial flowering parts of *C. calcitrapa*. Proximate analysis indicated a nutritional value of 145.45 ± 10.03 kcal/100 g, with a high content of organic matter (87.8 ± 1.13%) and moisture (63.27 ± 3.05%). The plant contained crude fiber (25.47 ± 1.94%), carbohydrates (17.61 ± 0.95 g/100 g), proteins (13.05 ± 1.94 g/100 g), fats (2.53 ± 0.2%), and free amino acids (5.29 ± 0.23 g/100 g). Ash analysis showed total ash (12.2 ± 1.13%), with notable water-soluble (10.12 ± 0.97%) and acid-soluble ash (9.85 ± 0.84%). Phytochemical screening indicated the presence of flavonoids, phenols, saponins, and moderate tannins, while quantitative analysis demonstrated high levels of total flavonoids (178.42 ± 19.81 mg QE/g) and phenolic content (112.85 ± 6.56 mg GAE/g), indicating strong phytochemical richness. Gas chromatography-mass spectrometry was used to analyze petroleum ether extract and identify 50 plant constituents, mostly fatty acids and hydrocarbons, with oleic acid (48.426%) and palmitic acid (15.834%) as the main constituents. HPLC analysis of the methanolic extract revealed 15 phenolic compounds, including rutin, apigenin, quercetin, kaempferol, epicatechin gallate, and protocatechuic acid. The methanolic extract exhibited strong antioxidant activity, with DPPH scavenging activity reaching 90.16 ± 1.13% at 1000 µg/mL and an IC_50_ of 24.01 ± 0.73 µg/mL, compared with 12.71 ± 0.7 µg/mL for ascorbic acid. The total antioxidant capacity showed an IC_50_ of 37.58 ± 1.21 µg/mL. The methanolic extract revealed moderate antibacterial activity against six human pathogenic bacteria, with inhibition zones ranging from 16.89 ± 0.95 to 20.98 ± 1.52 mm. MIC values ranged from 62.5 to 125 mg/mL, while MBC values ranged from 125 to 250 mg/mL. In addition, the methanolic extract exhibited in vitro inhibitory activity against α-amylase and α-glucosidase, with IC_50_ values of 37.26 ± 0.91 µg/mL and 20.19 ± 1.54 µg/mL, respectively. These results suggest the presence of bioactive compounds in the aerial flowering parts of *C. calcitrapa*. Overall, the methanolic extract demonstrated antioxidant and antibacterial activities, in addition to in vitro carbohydrate-hydrolyzing enzyme inhibitory activity. These findings indicate that *C. calcitrapa* may represent a promising source of bioactive constituents with potential applications in medicinal and nutritional fields.

## Introduction

In recent decades, the rapid emergence of antibiotic-resistant pathogens has become a major global health concern. The inappropriate and excessive use of antibiotics in human and veterinary medicine and agriculture has accelerated the development of multidrug-resistant microorganisms, reducing the effectiveness of many conventional antibiotics. Reports indicate that antibiotic resistance is increasing worldwide, with a significant proportion of bacterial infections now exhibiting resistance to commonly used antibiotics^1,2^. In addition to medical misuse, the intensive use of synthetic pesticides and antibiotics in agriculture contributes to the emergence of resistant plant pathogens and environmental pollution. Residues of these chemicals can accumulate in soil and aquatic ecosystems, posing risks to human health and biodiversity. Consequently, the growing problem of resistance and environmental pollution has spurred interest in safer and more sustainable alternatives^3^.

Diabetes is a chronic metabolic disease characterized by persistently high blood sugar levels, resulting from the body’s inability to produce enough insulin, its inability to use its own insulin production effectively, or both. Prolonged high blood sugar leads to serious complications affecting multiple organs, including the heart, kidneys, eyes, nerves, and blood vessels. Consequently, diabetes is one of the most serious global public health challenges of the twenty-first century^4^. The International Diabetes Federation (IDF) also estimates that around 589 million adults aged 20 to 79 had diabetes in 2024, representing about one in nine adults worldwide, and this number is expected to reach 853 million by 2050 if current trends continue^5^.

Recent years have witnessed a remarkable resurgence in scientific research on medicinal plants, given their potential to address current global health challenges and their status as safer and more cost-effective alternatives to manufactured drugs. Research in the field of herbal medicine continues to bridge traditional knowledge with modern pharmacological evidence, highlighting the importance of medicinal plants as direct therapeutic agents and as leading tools for discovering new medicines^6,7^. Medicinal plants have long been an integral part of human healthcare, serving as a primary source of therapeutic agents long before the advent of modern medicine. These plants contain a diverse array of bioactive phytochemicals, such as alkaloids, flavonoids, terpenoids, and phenolic compounds, responsible for a wide range of pharmacological activities, including antioxidant, antidiabetic, antimicrobial, anti-inflammatory, and anticancer properties^8–10^.

Plant extracts with high antioxidant capacity are rich in various biologically active compounds capable of scavenging free radicals, including phenolic compounds, terpenoids, flavonoids, and vitamins^11^. These phytochemicals play an important role in protecting biological systems from oxidative stress. Among them, plant-derived polyphenols have garnered significant attention due to their potent antioxidant activity. In many cases, polyphenols have demonstrated greater antioxidant activity than vitamins C and E in vitro, suggesting their potential importance in preventing oxidative damage in living systems^12^.

*Centaurea calcitrapa* L. (Asteraceae), commonly known as purple starthistle, is a biennial herbaceous plant widely distributed throughout Mediterranean Europe, North Africa, Western Asia, and parts of the Arabian Peninsula. It grows in diverse environments, including roadsides, agricultural fields, and sunny rocky slopes, and is characterized by its distinctive bright purple flower heads surrounded by spiny sheaths^13^. This species has a long history of ethnobotanical uses, traditionally used to treat fever, jaundice, gastrointestinal disorders, skin diseases and other ailments in folk medicine. Phytochemical studies have revealed that extracts of the *C. calcitrapa* plant contain a variety of bioactive compounds, including sesquiterpene lactones, flavonoids, and sterols, which have been associated with significant antioxidant, antimicrobial, and cytotoxic activities in recent studies^13^. Due to its diverse chemical composition and promising biological effects, *C. calcitrapa* is the subject of increasing pharmacological research aimed at validating its traditional uses and exploring its impact as a source of new therapeutic factors^14^. Therefore, it is essential to classify plant compounds based on their antioxidant and antibacterial properties^15^. These plant products offer significant advantages compared to manufactured chemicals, including higher efficacy, fewer side effects, lower cost, and greater accessibility^16^.

Although different parts of *C. calcitrapa* have been traditionally used in folk medicine, most previous scientific investigations have focused on its general phytochemical screening or selected biological activities, while comprehensive studies on the aerial flowering parts remain limited. In particular, no integrated study has simultaneously addressed the nutritional profile together with detailed metabolite characterization and multiple bioactivities of this specific plant part. Therefore, the present study aims to fill this gap by providing a comprehensive and integrated investigation of the aerial flowering parts of *C. calcitrapa*. This includes (i) nutritional composition analysis, (ii) phytochemical profiling using GC–MS, (iii) identification and quantification of phenolic compounds using HPLC, and (iv) evaluation of antioxidant, antidiabetic, and antibacterial activities of the methanolic extract. The novelty of this work lies in the combined application of nutritional analysis, advanced chromatographic techniques, and multi-target biological assays in a single study, offering a more holistic understanding of the chemical composition and bioactivity of this underexplored plant part. This integrated approach provides stronger scientific evidence of its pharmacological potential and establishes a basis for future isolation and characterization of its bioactive constituents.

## Material and methods

### Plant material

The aerial flowering parts of *Centaurea calcitrapa* L. were collected from Matrouh Port Road in Alexandria Governorate, Egypt, in March 2024. The species was identified by Professor Dr. Abd El-Halim Abd El-Mogali Mohamed, Chief Researcher, Flora and Phytotaxonomy Research Department, Horticulture Research Institute (HRI), Agricultural Research Center (ARC), Agricultural Museum, Dokki, Giza, Egypt. We prepared a reference specimen (CAIM-367) and deposited in the Herbarium of the Flora and Phytotaxonomy Department, HRI, ARC, Agricultural Museum, Dokki, Giza, Egypt. After collecting, the specimen was washed with distilled water and air-dried at room temperature until a constant weight was reached. The dried material was put in airtight bags for further analysis after being ground into a fine powder with an electric blender. All plant materials utilized in this study were collected and handled following institutional and national ethical and procedural guidelines under permission Code: AZU/SCI/BOT/ 2024-018.

### Preparation of extracts

The powdered aerial flowering parts of *C. calcitrapa* (500 g) were extracted using a Soxhlet apparatus with petroleum ether for 5 h at its boiling point (60 °C) using a heating mantle. The residue was then air-dried and further extracted with methanol for 8 h at its boiling point (75 °C) under continuous reflux conditions. The solvent ratio used was 500 g/5000 mL (w/v). After extraction, the obtained filtrates were concentrated under reduced pressure at 45 °C using a rotary evaporator. The resulting dried extracts were stored in the dark at 4 °C until further analysis. The petroleum ether extract was subjected to GC–MS analysis, while the methanolic extract was used for HPLC, antioxidant assays, antidiabetic and antibacterial activity evaluation^14^.

### Moisture content

The moisture content was determined according to^17^. A known weight of fresh plant material was dried in an oven at 40–60 °C for 6 h or until a constant weight was obtained. Moisture content was calculated as the percentage loss in weight relative to the initial fresh weight using the equation:

$${\text{Moisture }}\left( \% \right) \, = \, \left[ {\left( {{\text{Fresh weight}} - {\text{dry weight}}} \right)/\left( {\text{Fresh weight}} \right)} \right] \, \times {1}00$$

### Total ash

In brief, 5 g of the dried powdered material were burnt in a muffle furnace at 600 °C for 3 h before remaining to cool^18^. The following formula was used to calculate the ash percentage after the residual ash was weighed:

$$\begin{aligned} &{\text{Ash }}\left( \% \right) \, = \, \left[ {\left( {\text{Weight of ash after incineration}} \right)/\left( {\text{Initial weight of sample}} \right)} \right] \, \times {1}00\\ & {\text{The organic matter content }} = \, \left( {{\text{Weight of crucible }} + {\text{ plant sample}}} \right) \\ &- \left( {{\text{Weight of empty crucible }} + {\text{ plant ash}}} \right)\\ & {\text{Organic matter }}(\% )\, = \,\left[ {\left( {\text{Organic matter weight}} \right)/\left( {\text{Dry sample weight}} \right)} \right]\, \times \,{1}00\end{aligned}$$

### Acid-insoluble ash

Acid-insoluble ash was determined by adding 25 mL of 2 M hydrochloric acid to the total ash, covering the mixture with a watch glass, and heating it gently for 5 min. The mixture was then filtered through ash-free filter paper, and the residue was washed with hot water until neutral. The acid-insoluble residue was dried to constant weight, cooled, and weighed. The obtained residue represents the acid-insoluble ash^17^.

$$\begin{aligned} & {\mathrm{Acid}} - {\text{insoluble ash }}\left( \% \right) \\ & \quad = \, \left[ {\left( {{\text{Weight of acid}} - {\text{insoluble residue}}} \right)/\left( {{\text{Weight of air}} - {\text{dried sample}}} \right)} \right] \, \times { 1}00 \\ & {\mathrm{Acid}} - {\text{soluble ash }}\left( \% \right) \, \\ & \quad = \, [\left( {{\text{Total ash}} - {\text{Acid insoluble ash}}} \right)/({\text{Weight of air}} - {\text{dried sample}})] \, \times { 1}00 \\ \end{aligned}$$

### Water-insoluble ash

The total ash was combined with 25 mL of distilled water and gently boiled for 5 min. The resulting mixture was then filtered through ash-free filter paper. The residue collected on the filter paper was washed with hot water, transferred to a pre-weighed crucible, dried, ignited, and weighed. This final residue constitutes the water-insoluble ash^17^.

$$\begin{aligned} & {\mathrm{Water}} - {\text{insoluble ash }}\left( \% \right) \\ & \quad = \, \left[ {\left( {{\text{Weight of water}} - {\text{insoluble residue}}} \right)/\left( {{\text{Weight of air}} - {\text{dried sample}}} \right)} \right] \, \times { 1}00 \\ & {\text{Water soluble ash }}\left( \% \right) \, \\ & \quad = \, [\left( {{\text{Total ash}} - {\text{Water insoluble ash}}} \right)/({\text{Weight of air}} - {\text{dried sample}})] \, \times { 1}00 \\ \end{aligned}$$

### Total lipid

The lipid matter of *C. calcitrapa* was examined using the Soxhlet extraction process as defined by Arunachalam et al.^19^. Take 10 g of dry powdered was continuously extracted over the course of 6 h utilizing 250 mL of petroleum ether (60–80 °C). Evaporation at 102 °C was used to concentrate the resultant solvent extract until a consistent weight was reached. Following cooling in a desiccator, the residue was weighed and the subsequent formula was used to calculate the crude lipid percentage:

$$\begin{aligned} & \% {\text{ Lipid }} = \, \big[ \big( {{\text{Weight of the bottle with the fat residue }}{-}{\text{ Weight of empty bottle}}}\big)\\ &/\big( {\text{Weight of sample}}\big) \big] \, \times {1}00 \end{aligned}$$

### Crude fiber

To determine the crude fiber content, the standard acid–base digestion method was used. Two grams of the defatted vegetable powder sample were added to 200 mL of 1.25% sulfuric acid solution, and the mixture was heated under reflux for 30 min. After filtration, the sample was washed with distilled water until neutralization. The insoluble material was added to 200 mL of 1.25% sodium hydroxide solution, and the mixture was heated under reflux for 30 min. After filtration, the sample was successively washed with hot distilled water and ethanol. The extracted residue was dried to steady weight at 105 °C and then incinerated at 550 °C. The crude fiber content was calculated based on the weight of the residue after incineration^20,21^. The crude fiber percentage was calculated using the following equation:

$${\text{Crude fiber }}\left( \% \right) \, = \, \left[ {\left( {{\mathrm{W}}_2 \, - {\text{ W}}}_1 \right) /{\text{Weight of dry sample}}} \right] \, \times { 1}00$$

where: W_2_ = Weight of dried residue (g), W_1_ = Weight of ash after incineration (g).

### Soluble protein content

Protein content was estimated using the Lowry colorimetric method. Briefly, 100 mg of the powdered sample was homogenized in 10 mL of 0.2 M phosphate buffer, and the sample was centrifuged at approximately 900×*g* (RCF) using an 80–1 electronic centrifuge for 10 min. One mL of the filtrate was collected, and 5 mL of basic copper reagent was added. The mixture was left to stand for 10 min. Subsequently, 0.5 mL of 1 M Folin-Cioalto reagent was added, and the mixture was left to react in the dark for 30 min. The absorbance was measured at 660 nm using a spectrophotometer^22^. It should be noted that the Lowry assay provides an estimate of soluble protein content and does not represent total nutritional protein content as determined by nitrogen-based methods such as Kjeldahl or Dumas analysis.

### Total free amino acids

The total free amino acid concentration has been determined using^23,24^ A 1 mL of amino acid extract was mixed with 1 mL of ninhydrin reagent. Then, 5 mL of the diluted solvent were added to all test tubes and the mixture was left at room temperature for 15 min. The absorbance of the resulting solution was then measured at 570 nm.

### Total carbohydrates

The total carbohydrate content was determined using the anthron method described by Chandran et al.^25^. Briefly, 100 mg of the sample powder was hydrolyzed with 5 mL of 2.5 M hydrochloric acid in a boiling water bath for 3 h. The solution was then neutralized with sodium carbonate, diluted to 100 mL, and filtered. The filtrate was centrifuged at approximately 900×*g*(RCF) for 10 min. One mL of the supernatant was mixed with 4 mL of anthron reagent, heated in a boiling water bath for 8 min, and then cooled. The absorbance was measured at 630 nm using a spectrophotometer. The nutritional value was subsequently confirmed according to the method of Indrayan et al.^26^.

### Determination of extractive value

Take 5 g of the dried plant powder extracted over 24 h using 100 mL of a suitable solvent (ether, methanol, or water). The mixtures were shaken and left to stand overnight, and the solvents were recovered by precipitation and filtration. The filtrate was dried, and the extraction yield was calculated as a percentage of the initial plant material^27^.

$${\text{Extract }}\left( \% \right) \, = \, \left[ {\left( {\text{Weight of the extract obtained}} \right)/\left( {\text{Weight of sample taken}} \right)} \right] \, \times {1}00$$

### Qualitative of phytochemical constituents

A qualitative phytochemical screening of the dried powder was carried out following the methods described by^28–32^.

### Quantitative of phytochemical constituents

#### Total phenolic content

Total phenolic content was estimated using the Folin–Ciocalteu method^33,34^. Briefly, 50 μL of extract (1 mg/mL) or standard solution was mixed with 500 μL of Folin–Ciocalteu reagent (1 M) and 950 μL of distilled water. After 5 min, 2.5 mL of 5% Na_2_CO_3_ solution was added. The mixture was incubated in the dark for 40 min, and the absorbance was measured at 725 nm.

### Total tannins

Total tannin content was assessed using the Folin–Denis spectrophotometric method described by^35,36^. An aliquot of 0.5 mL extract and 0.5 mL distilled water were added to 100 mg of polyvinylpolypyrrolidone (PVPP). The tubes were incubated at 4 °C for 4 h and then centrifuged approximately 900×*g* (RCF) for 10 min at 4 °C. The supernatant contained only non-tannin phenolic compounds. Subsequently, 100 μL of this supernatant and 0.5 mL of Folin–Ciocalteu reagent (1 M) were mixed in triplicate and incubated for 5 min. Then, 2.5 mL of 5% sodium carbonate solution was added. The mixture was vortexed and incubated in the dark for 40 min. Absorbance was measured at 725 nm using a UV–Vis spectrophotometer.

### Total flavonoids

Total flavonoid content was determined following the method of^37^. Briefly, 500 μL of extract (1 mg/mL) was mixed with 150 μL of 5% NaNO_2_ and 500 μL of distilled water. After 5 min of incubation at room temperature, 150 μL of 10% AlCl_3_ was added, followed by 2 mL of 4% NaOH and distilled water to a final volume of 5 mL. The mixture was incubated at 40 °C for 15 min, and absorbance was measured at 510 nm.

### Total flavonols

Total flavonol content was measured using the AlCl_3_ colorimetric technique outlined by^38^. Briefly, 500 μL of extract was combined with 6 mL of sodium acetate (CH_3_COONa) solution and 2 mL of 2% AlCl_3_. The mixture was incubated for 2 h at room temperature, and absorbance was measured at 440 nm.

### Total alkaloids

Total alkaloid content was determined according to the method of^39^. Five grams of dried sample were mixed with a 1:4 (v/v) mixture of glacial acetic acid and 70% ethanol. After 4 h of incubation, the mixture was filtered. Concentrated ammonia solution was added to the filtrate to precipitate the alkaloids. The precipitate was collected by filtration through pre-weighed filter paper, and the alkaloids were dried in an oven at 70 °C until a constant weight was achieved.

### Total saponins

Total saponin content was estimated following the method described by^40,41^. Five grams of sample were extracted twice with 100 mL of 20% ethanol at 55  °C. The combined extracts were concentrated to 40 mL at 90 °C. The concentrate was partitioned with 40 mL of diethyl ether, and the aqueous layer was further extracted with 60 mL of n-butanol. The butanol fraction was washed with 5% sodium chloride solution and then evaporated to dryness at 60 °C until a constant weight was obtained.

### Total steroids

Total steroid content was analyzed following^39^. Five grams of sample were boiled in 50 mL of hydrochloric acid for 30 min, then filtered and extracted with ethyl acetate. The organic layer was dried, treated with concentrated amyl alcohol, and heated at 100 °C for 5 min. The resulting cloudy extract was filtered, cooled in a desiccator, and weighed.

### Phytates

Phytate content was determined using the method of^42^. Four grams of each sample were immersed in 100 mL of 2% hydrochloric acid for 5 h and then filtered. A 25 mL aliquot of the filtrate was transferred to a conical flask, and 5 mL of 0.3% ammonium thiocyanate indicator solution was added. The pH was adjusted to 3.5 by adding 53.5 mL of distilled water. The mixture was titrated with ferric chloride solution until a stable yellow–brown color persisted for 5 min.

### Oxalates

Oxalate content was determined according to^42^. One gram of each powdered sample was mixed with 75 mL of 3.0 M sulfuric acid and stirred continuously for approximately 1 h using a magnetic stirrer. The mixture was then filtered. A 25 mL aliquot of the filtrate was titrated with 0.05 M potassium permanganate solution at 80 °C until a light pink color persisted for at least 30 s.

### Cyanogenic glycosides

Cyanogenic glycoside content was estimated following^42^. Four grams of dried sample were mixed with 2 mL of orthophosphoric acid and 40 mL of distilled water and allowed to stand for 24 h to release bound hydrogen cyanide. The extract was distilled with paraffin (to prevent foaming) and paraffin flakes (to prevent effervescence). A 5 mL aliquot of the distillate was collected in a flask containing 0.1 g of sodium hydroxide in 40 mL of water and then diluted to 50 mL. A 20 mL portion of this diluted distillate was mixed with 1 mL of 5% potassium iodide solution and titrated with 0.01 M silver nitrate solution until a slight, persistent turbidity appeared.

### GC–MS analysis

An TG–5MS capillary column (30 m × 0.25 mm × 0.25 µm) and a Trace GC-TSQ mass spectrometer (Thermo-Scientific) were used to analyze volatile constituents. After maintaining the oven temperature at  50 °C, it ramped up to 250 °C (2 min hold) at 5 °C/min and then increased to 300 °C at 30 °C/min (2 min hold). At 270 °C and 260 °C, respectively, the injector and MS transfer line were set. A carrier gas of helium (1 mL/min) was employed. One-microliter samples were injected in split mode using an Autosampler AS1300. Electron impact (EI) spectra were acquired at 70 eV over m/z 50–650, with the ion source at 200 °C^43^. The chemical constituents were tentatively identified by comparing their mass spectra and retention times with those available in the WILEY 09 and NIST 14 mass spectral libraries. The relative abundance of each compound was estimated from its peak area percentage in the total ion chromatogram. As retention indices and authentic reference standards were not determined in the present study, compound identification was based on library matching and retention time comparison and should therefore be regarded as tentative.

### HPLC analysis

Using an Agilent 1260 Infinity II system with a diode-array detector and an Eclipse XDB-C18 column (150 × 4.6 mm, 5 µm) with a C18 guard column, polyphenols were analyzed by HPLC^44^. The mobile phase consisted of 2% aqueous acetic acid (B) and acetonitrile (A) at a gradient of 0.8 mL/min for 60 min. Benzoic acid derivatives were detected at 280 nm, flavonoids at 360 nm, and derivatives of cinnamic acid at 320 nm. Prior to injection, a 0.45 µm syringe filter was used to filter the 50 µL of samples. Compounds were identified by comparing retention periods and UV spectra to standards.

### Antioxidant assays

#### DPPH

The DPPH was evaluated using the method of Cromwell and Afolayan^45^. A freshly made DPPH radical methanol solution (0.004%, w/v) was prepared and kept in the dark at 10 °C. A 100 μL (1.95 to 1000 µg/mL) of methanolic extract was combined with 3 mL of the DPPH solution. Additionally, a control was created using only methanol and DPPH solution. After thoroughly vortexing the reaction mixture, it was allowed to stand for 30 min in the dark. Absorbance at 517 nm was recorded against methanol.

$$\% {\text{ DPPH Scavenging }} = \, \left[ {\left( {{\mathrm{A}}_{\mathrm{o}} \, - {\text{ A}}}_{\mathrm{s}} \right) /{\mathrm{A}}}_{\mathrm{o}} \right] \, \times { 1}00$$

where A_o_ is the control absorbance and A_s_ is the sample absorbance.

#### TAC

Total antioxidant capacity was assessed by^46^. In test tubes, 0.3 mL of the methanolic extract and standard medications (12.5 to 400 µg/mL) were dissolved in 3 mL of the reagent solution (0.6 M sulphuric acid, 4 mM ammonium molybdate, and 28 mM sodium phosphate). The tubes were covered and left in a water bath at 95 °C for 95 min. After allowing the mixture to reach room temperature, the absorbance at 795 nm was measured. In place of the samples, a mixture containing distilled water served as the control. The inhibition percentage was calculated as follows:

$$\% {\text{ Total antioxidant activity}} = \, \left( {{\mathrm{A}}_{\mathrm{s}} \, - {\text{ A}}}_{\mathrm{o}} \right) /{\mathrm{A}}_{\mathrm{s}} \, \times { 1}00$$

where A_s_ is the sample absorbance and A_o_ is the control absorbance.

### Antibacterial activity

Various pathogenic strains were tested to explore the antibacterial potential of the methanol extract from the *C. calcitrapa*. These included Gram-positive bacteria; *Staphylococcus haemolyticus* (ATCC 29970), *Staphylococcus aureus* (ATCC 25923), and *Bacillus subtilis* (ATCC 6633), Gram-negative bacteria; *Klebsiella pneumoniae* (ATCC 2146), *Escherichia coli* (ATCC 8739), and *Acinetobacter baumannii* (ATCC 19606). To assess effectiveness, the well-diffusion method in agar was used^47^. All bacterial species used in this study were cultured on nutrient agar (NA) and incubated at 37 °C for 48 h. The cultures were inoculated onto 15 cm diameter nutrient agar plates after adjusting their concentration according to the McFarland turbidity standard of 0.5. The methanolic extract was dissolved in dimethyl sulfoxide (DMSO) to obtain the desired concentrations. Three wells (6 mm diameter) were punched into the agar using a sterile cork borer. One well was filled with 100 μL of the methanolic extract, one with ciprofloxacin as a positive control, and one with DMSO as a negative (solvent) control. The plates were allowed to stand for 1 h to facilitate diffusion and then incubated at 35 ± 2 °C for 24 h. Antibacterial activity was determined by measuring the diameter of the inhibition zones (mm). All experiments were performed in triplicate.

### Minimum inhibitory concentrations (MICs) and minimum bactericidal concentrations (MBCs)

Both MIC and MBC were determined using the broth microdilution method with minor modifications to CLSI guidelines^48^. The methanolic extract was dissolved in DMSO to prepare a stock solution (1000 mg/mL) and subsequently diluted with Mueller–Hinton broth (MHB), ensuring that the final concentration of DMSO did not exceed 1% (v/v). Two-fold serial dilutions were prepared to obtain final concentrations ranging from 62.5 to 250 mg/mL. In sterile 96-well microplates, 100 µL of each dilution was mixed with 100 µL of bacterial suspension (approximately 5 × 10^5^ CFU/mL). Control wells included a growth control (broth + inoculum), a sterility control (broth only), a solvent control (broth + inoculum + 1% DMSO), and a positive control (gentamicin, 10 µg/mL). The plates were incubated at 37 °C for 18–24 h. MIC was defined as the lowest concentration exhibiting no visible bacterial growth. For MBC determination, aliquots (10–100 µL) from wells showing no visible growth were subcultured onto drug-free Mueller–Hinton agar plates and incubated at 37 °C for 24 h. The MBC was defined as the lowest concentration showing no colony formation. All experiments were performed in triplicate.

### α-Amylase inhibition test

Alpha-amylase inhibitory activity was determined using the 3,5-dinitrosalicylic acid (DNSA) method^49^. Plant extracts were prepared in 10% DMSO and phosphate buffer (0.02 M Na_2_HPO_4_/NaH_2_PO_4_, 0.006 M NaCl, pH 6.9) at concentrations ranging from 1.9 to 1000 µg/mL. Briefly, 200 µL of the extract was incubated with 200 µL of alpha-amylase solution (2 units/mL) at 30 °C for 10 min. Subsequently, 200 µL of starch solution (1% w/v) was added, and the mixture was incubated for another 3 min. The reaction was stopped by adding 200 µL of DNSA reagent, and the mixture was heated in a boiling water bath at 85–90 °C for 10 min. After cooling, the mixture was diluted with 5 mL of distilled water, and the absorbance was measured at 540 nm. A control sample (enzyme + buffer solution) and a blank sample (extract without enzyme) were prepared simultaneously. The inhibition rate was calculated using the following formula, with the IC_50_ value extracted from the inhibition curve:

$$\% \, \alpha {\text{-Amylase Inhibition }} = \, \left( {{\mathrm{A}} _{\mathrm{o}}\, - {\text{ A}}}_{\mathrm{s}} \right) /{\mathrm{A}}_{\mathrm{o}} \, \times { 1}00$$

where A_o_ is the absorbance of the control group (100% enzyme activity), and A_s_ is the absorbance of the sample.

### α-Glucosidase inhibition activity test

Alpha-glucosidase inhibition activity was determined according to standard methods^50,51^. The test sample was dissolved in dimethyl sulfoxide (DMSO). The enzyme solution (0.8 units/ml of α-glucosidase, dissolved in 50 mM phosphate buffer, pH 7.0, containing 100 mM sodium chloride) and the substrate (0.7 mM p-nitrophenyl-α-D-glucopyranoside (pNPG)) were freshly prepared. The experimental procedure was as follows: 20 µL of the sample was incubated with 80 µL of the enzyme solution at 37 °C for 5 min. Then, 1.9 mL of the substrate solution was added, and the incubation continued for 15 min. The reaction was stopped by adding 2 mL of 0.5 M Tris solution, and the resulting para-nitrophenol was measured at 400 nm absorbance. Dimethyl sulfoxide (DMSO) was used as a negative control, and acarbose was used as a positive control. The inhibition rate was calculated as follows:

$$\%\, \alpha{\text{-GlucosidaseInhibition }} = \, \left( {{\mathrm{A}} _{\mathrm{o}}\, - {\text{ A}}}_{\mathrm{s}} \right) /{\mathrm{A}} _{\mathrm{o}}\, \times { 1}00$$

where A_o_ is the absorbance of the control group (100% enzyme activity), and A_s_ is the absorbance of the sample.

### Statistical analysis

Statistical analysis was performed using Microsoft Excel 365 and Minitab® 19. The data were assumed to follow a normal distribution. Results are presented as mean ± standard deviation (SD), with a 95% confidence interval and a significance level of 5% (*P* < 0.05). One-way analysis of variance (ANOVA) and Tukey’s HSD post-hoc test were used to compare data from multiple groups. An independent samples t-test was also used to compare data from two groups. A *P* < 0.05 was considered statistically significant.

## Results and discussion

### Proximate composition of the aerial flowering parts of *C. calcitrapa*

As shown in Table 1, the proximate chemical composition and nutritive value of the plant under study are presented. The result exhibited a nutritive value of 145.45 ± 10.03 kcal/100 g, with organic matter constituting 87.8 ± 1.13%, while the moisture content was 63.27 ± 3.05%. Additionally, the composition includes crude fiber (25.47 ± 1.94%), total lipid (2.53 ± 0.2%), total carbohydrates (17.61 ± 0.95 g glucose/100 g), protein content, as estimated by the Lowry assay (13.05 ± 1.94 g BSA/100 g), total free amino acids (5.29 ± 0.23 g leucine/100 g). The contents of total ash, water-soluble ash, acid-soluble ash, acid-insoluble ash, water-insoluble ash were 12.2 ± 1.13%, 10.12 ± 0.97%, 9.85 ± 0.84%, 2.35 ± 0.3%, 2.09 ± 0.16%, respectively.

**Table 1:** Proximate chemical composition of the aerial flowering parts of *C. calcitrapa.*

Components	Result
Moisture (%)	63.27 ± 3.05
Organic matter (%)	87.8 ± 1.13
Ash (%)	12.2 ± 1.13
Acid soluble ash (%)	9.85 ± 0.84
Acid insoluble ash (%)	2.35 ± 0.3
Water soluble ash (%)	10.12 ± 0.97
Water insoluble ash (%)	2.09 ± 0.16
Total lipid (%)	2.53 ± 0.2
Crude fiber (%)	25.47 ± 1.94
Soluble protein content (g BSA/100 g)	13.05 ± 1.94
Total free amino acid (g leucine/100 g)	5.29 ± 0.23
Total carbohydrate (g glucose/100 g)	17.61 ± 0.95
Nutritive value (Kcal/100 g)	145.45 ± 10.03

The data are presented as mean ± standard deviation (n = 3). BSA, Bovine serum albumin.

In this study, the fresh aerial flowering parts of *C. calcitrapa* have a high-water content, which is common for medicinal plants that contain a lot of polar constituents. *Centaurea raphanina* has also been observed to exhibit similar moisture levels in dried samples, as have other Asteraceae medicinal plants^52,53^

The high level of organic matter is a reflection of the high level of structural and secondary metabolite biosynthesis. Similar findings have been reported in several species of the genus *Centaurea*, such as *Centaurea raphanina* and *Centaurea melitensis*, where high organic matter content has been associated with enhanced biological activities, including antimicrobial and antioxidant properties^53,54^.

The nutritional value of medicinal plants offers valuable insights into their potential uses in food preparations and functional medicines. The nutritional value of plant material is reflected in its total content of fats, proteins, and carbohydrates^55,56^. The findings of *C. calcitrapa* align well with earlier reports^57,58^.

The aerial flowering parts of *C. calcitrapa* showed an intermediate total ash content (12.2 ± 1.13%), with a higher proportion of water-soluble and acid-soluble ash. This suggests that most of the mineral content is readily available for food and pharmaceutical purposes. These results are consistent with previous reports on other medicinal plants, which indicate increased bioavailability of essential ions^59,60^. The low acid-insoluble ash and water-insoluble ash suggests minimal contamination with soil. Similar trends were observed in other Asteraceae species, including *Eclipta alba* and *Pulicaria incisa subsp. Incisa*^61,62^. The crude fiber content was notably high that associated with both nutritional benefits and potential health. These finding is aligned with previous findings reported for aerial parts of *Centaurea raphanina*^53^. In the genus *Centaurea*, lipid analysis has been used to estimate the nutritional value and functional potential of the aerial parts, as these plants are known to accumulate biologically active fatty acids, esters, and lipophilic secondary metabolites, which contribute to their therapeutic value^63,64^.

Also, the dried aerial flowering parts of *C. calcitrapa* indicate the presence of an energy reserve in the plant material. Similar carbohydrate levels have been recorded in various species of the genus *Centaurea*, where carbohydrates have been found to contribute significantly to their antioxidant and antidiabetic properties. Therefore, the current findings are consistent with previous studies conducted on members of the Asteraceae family^65–67^. The soluble protein content determined by the Lowry method is comparable to that reported for other *Centaurus* species^67^. This supports the nutritional importance of the plant and its potential role in therapeutic applications, including anti-cancer and anti-inflammatory activities^68^.

Total free amino acids (TFAAs) are essential contributors to plant metabolism, stress responses, and the biosynthesis of pharmacologically active secondary metabolites, including alkaloids, flavonoids, and phenolic content. As such, they enhance the therapeutic potential of herbal preparations^69,70^.

### Extractive value obtained from the dried aerial flowering parts of *C. calcitrapa*

**Fig. 1:**
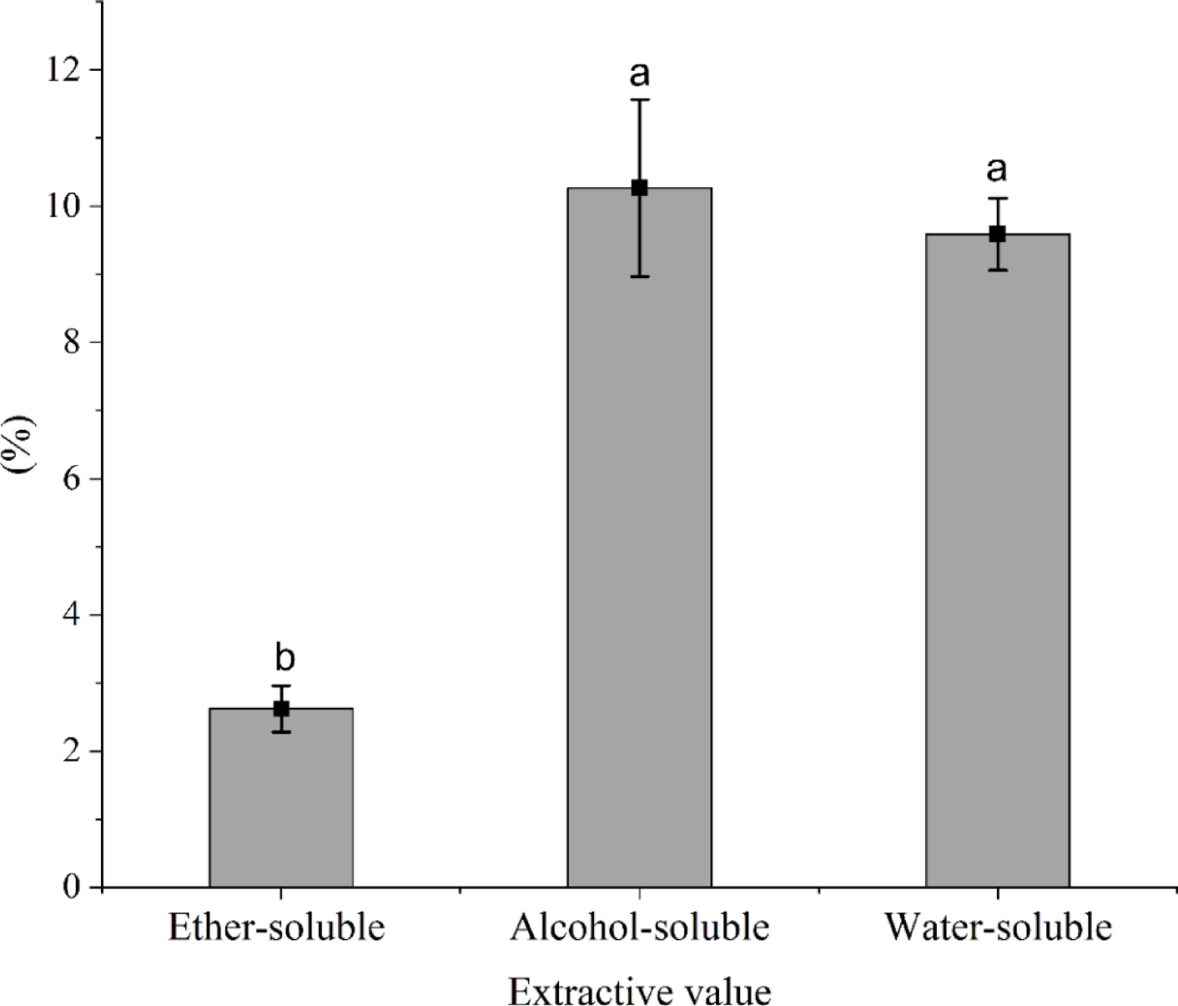
Extractive value obtained from the dried aerial flowering parts of *C. calcitrapa* by different solvents. Data in the columns are expressed as mean ± standard deviation (n = 3).

The extractive yield obtained from the dried aerial flowering parts of *C. calcitrapa* using different solvents are presented in Fig. 1. The highest yield was achieved with alcohol (10.27 ± 1.3%) followed by water-soluble (9.59 ± 0.53%) and ether-soluble (2.62 ± 0.34%).

Extraction value measures the amount of biologically active compounds that can be extracted from plant material using different solvents, such as water, alcohol, or ether. It reflects the solubility and availability of phytochemicals responsible for the therapeutic, antioxidant, and antimicrobial properties of medicinal plants^58,71^. Alcoholic extraction value is a critical criterion in the evaluation of medicinal plants, as alcoholic solvents can dissolve a wide range of polar and semi-polar phytochemicals. In the present study, the highest extractive value was obtained using alcohol, indicating that the flowering aerial parts of *C. calcitrapa* are rich in alcohol-soluble bioactive constituents, which compounds are recognized for their antioxidant, antimicrobial, and anti-inflammatory activities. This result was closely related to^14^.

The value of the aqueous extraction reflects the presence of highly polar components, such as sugars, amino acids, inorganic salts, water-soluble phenols and some glycosides, reflecting the traditional method of consumption. In this study, the aqueous extractive value ranked second after alcohol, indicating abundance of water-soluble constituents in *C. calcitrapa* that may contribute to its nutritional and therapeutic activities. This finding aligns with^72,73^.

The value of the ether extract represents the proportion of nonpolar, lipophilic compounds, such as lipids, waxes, sterols, terpenoids, and hydrocarbons. These compounds are often associated with membrane stability and certain antimicrobial and anti-inflammatory properties^74^. In the present study, the lowest extractive value was obtained with ether, indicating a lower content of non-polar constituents in *C. calcitrapa*. This pattern is consistent with previous studies on *C. calcitrapa*^14^*.*

### Preliminary phytochemical screening of *C. calcitrapa*

**Fig. 2:**
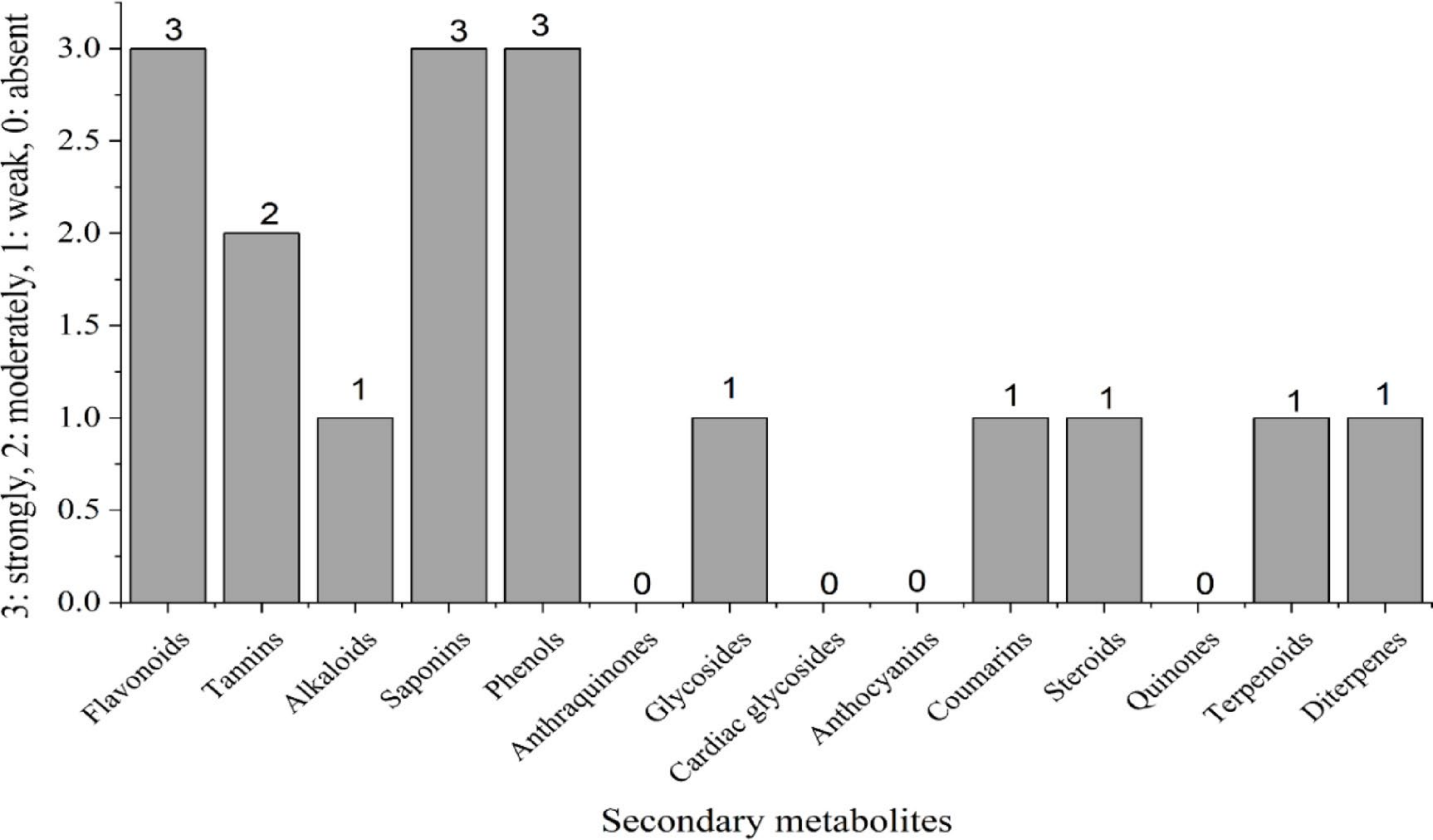
Preliminary phytochemical screening of the dried aerial flowering parts of *C. calcitrapa.*

As shown in Fig. 2, preliminary phytochemical analysis of the dried flowering aerial parts of *C. calcitrapa* revealed several important secondary metabolites, albeit at varying concentrations. Flavonoids, saponins, and phenolic compounds were found at high concentrations, while tannins were found at moderate concentrations. Alkaloids, glycosides, coumarins, steroids, terpenes, and diterpenes were found at low concentrations. In contrast, cardiac glycosides, anthocyanins, quinones, and anthraquinones were not detected based on the tests performed.

Primary phytochemical screening is a qualitative analysis used to identify the major classes of secondary metabolites found in medicinal plants. These metabolites such as flavonoids, phenols, alkaloids, terpenoids, saponins, and glycosides are widely recognized for their critical functions in plant defense and their contributions to a range of pharmacological activities, including antioxidant, antimicrobial, anti-inflammatory, and antidiabetic effects^75^. In the current investigation, the aerial flowering parts of *C. calcitrapa* demonstrated high concentrations of saponins, flavonoids, and phenols. Flavonoids are renowned for their robust antioxidant properties and their capacity to suppress oxidative stress, while saponins offer antibacterial and cell-protective effects. Phenolics enhance antioxidant capacity and are associated with anti-inflammatory and cardioprotective properties^76,77^. These finding align with previous report^14,32,78^.

### Quantitative analysis of phytochemical and anti-nutrient composition

The quantitative phytochemical analysis of the aerial flowering parts of *C. calcitrapa* is summarized in Table 2. The results revealed total flavonoid, flavonol, phenolic, and tannin contents of 178.42 ± 19.81 mg QE/g, 38.36 ± 3.42 mg RTE/g, 112.85 ± 6.56 mg GAE/g and 32.5 ± 2.29 mg TAE/g, respectively. While total alkaloids, saponins and steroids, oxalates, phytates and cyanogenic glycosides were 1.31 ± 0.27 mg/100 g, 3.8 ± 0.53 mg/100 g,1.43 ± 0.18 mg/100 g, 25.83 ± 1.54 mg/100g, 3.92 ± 0.13 mg/100g, 12.24 ± 1.22 mg/100g, respectively.

**Table 2:** Quantitative analysis of phytochemical and anti-nutrient composition of the dried aerial flowering parts of *C. calcitrapa.*

Items	Result	Unit
Phenolic content	112.85 ± 6.56	mg GAE/g DW
Tannins	32.5 ± 2.29	mg TAE/g DW
Flavonoids	178.42 ± 19.81	mg QE/g DW
Flavanols	38.36 ± 3.42	mg RTE/g DW
Alkaloids	1.31 ± 0.27	mg/100 g DW
Saponins	3.8 ± 0.53	mg/100 g DW
Steroids	1.43 ± 0.18	mg/100 g DW
Oxalates	25.83 ± 1.54	mg/100 g DW
Phytates	3.92 ± 0.13	mg/100 g DW
Cyanogenic glycosides	12.24 ± 1.22	mg/100 g DW

The data are presented as mean ± standard deviation (n = 3).

Quantitative analysis of the dried aerial flowering parts of *C. calcitrapa* revealed a remarkable abundance of bioactive phytochemicals and low levels of antinutrients, supporting the plant’s potential nutritional and medicinal value. Phenolics and flavonoids were among the most abundant constituents, with contents of 112.85 ± 6.56 mg GAE/g and 178.42 ± 19.81 mg QE/g, respectively (Table 2). These high levels are consistent with the strong positive qualitative presence of phenolic compounds previously found and confirm the role of *C. calcitrapa* as a rich source of phenolic antioxidants. The high content of phenols and flavonoids in plant extracts has been widely attributed with potent antioxidant, anti-inflammatory, and antimicrobial properties, as has been reported in other species of the medicinal Asteraceae family such as *Centaurea hypoleuca* and *C. raphanina*^53,79,80^.

The amount of tannins was determined to be 32.5 ± 2.29 mg TAE/g, and flavanols to be 38.36 ± 3.42 mg RTE/g, indicating the presence of polymeric phenolic subgroups that may contribute to antimicrobial and enzyme-modifying activities^81,82^. Tannins are also involved in defense mechanisms against pathogens in plants and can provide therapeutic benefits such as anti-inflammatory activity^83^. Saponins have been reported to enhance cholesterol metabolism and immune effects. Alkaloids, even at low concentrations, may contribute to analgesic and anti-disease properties, as supported by recent phytochemical investigations^84^.

Oxalates were found at a concentration of 25.83 ± 1.54 mg/100 g, while phytates were present at a low concentration of 3.92 ± 0.13 mg/100 g, indicating a minimal impact on mineral bioavailability. Similarly, cyanogenic glycosides were measured at 12.24 ± 1.22 mg/100 g, suggesting a moderate potential risk, but remaining within ranges considered safe for many herbal applications when properly processed. Previous research confirms that plants containing similar levels of antinutrients can be suitable for nutritional and therapeutic use after being properly prepared^81,85,86^.

### GC–MS analysis

**Fig. 3:**
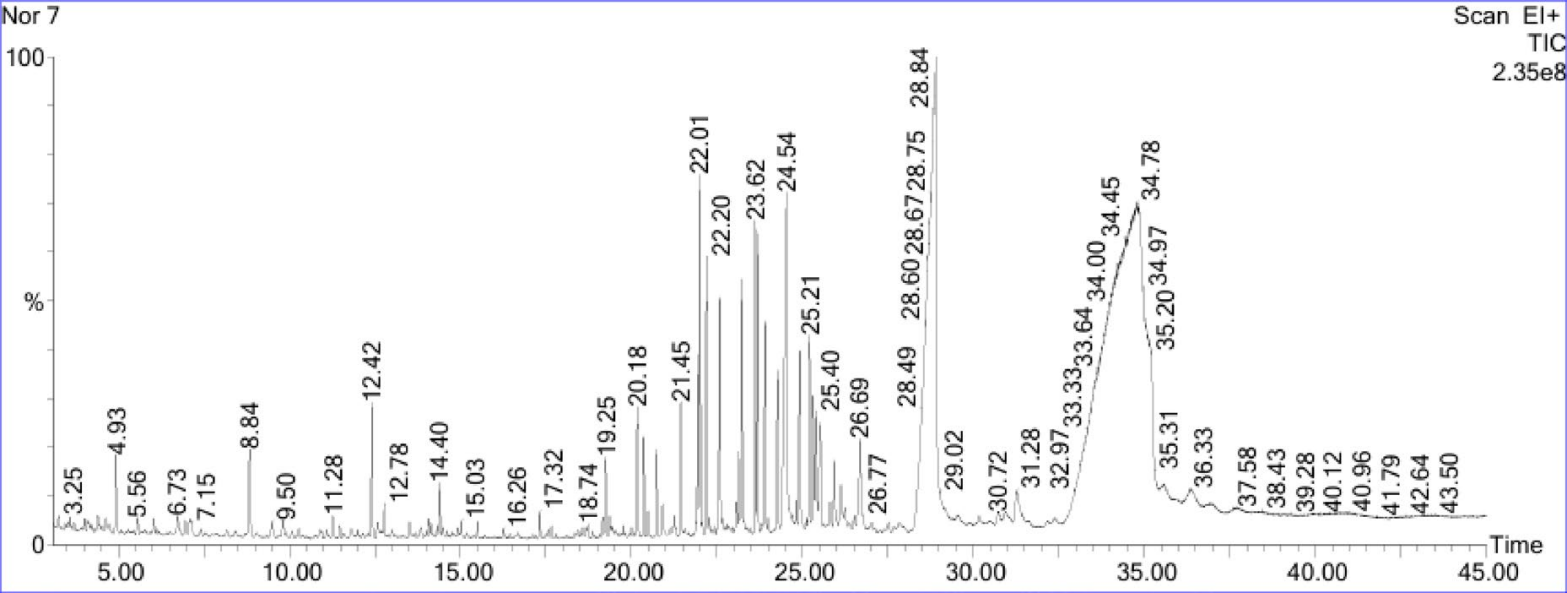
GC–MS chromatogram of *C. calcitrapa* petroleum ether extract.

GC–MS analysis of the petroleum ether extract of *C. calcitrapa* revealed a chemically diverse profile comprising 50 phytoconstituents (Fig. 3 & Table 3). The compounds detected were mostly hydrocarbons, fatty acids, fatty acid esters, terpenoids, and aromatic derivatives, reflecting the nonpolar nature of the extraction solvent. Aliphatic hydrocarbons were widely represented, including decanes, dodecanes, tridecanes, pentadecanes, octadecanes, henicosanes, and tricosanes, although each was found in relatively low proportions (< 1%). Several alkyl-substituted benzene derivatives were also identified, which together contributed a significant portion of the extract. Compounds tentatively assigned to fatty acids constituted the dominant chemical class in the chromatographic profile. The compound tentatively identified as oleic acid represented the largest chromatographic peak (48.426% relative peak area), followed by n-hexadecanoic acid (palmitic acid) (15.834%). Other fatty acids detected in smaller amounts included tetradecanoic acid (3.149%), heptadecanoic acid (0.204%), pentadecanoic acid (0.294%), hexadecenoic acid (*Z*-11-) (0.183%), and 9,12-octadecadienoic acid (*Z,Z*)- methyl ester (0.129%). Additionally, 9,12,15-octadecatrienoic acid glycerol ester (0.169%) was recorded. Among the terpenoid-related compounds, phytol (0.561%) and geranyl isovalerate (0.448%) were identified, along with 4*α*,7,7,10*α*-tetramethyldodecahydrobenzo[*f*]chromine-3-ol (2.041%), which may link to the biological activities of the extract. The diterpene alcohol phytol and long-chain polyunsaturated fatty acids are known for their antimicrobial and antioxidant properties. The predominance of unsaturated fatty acids, particularly oleic acid, indicates that the petroleum ether extract is rich in lipophilic bioactive components. Overall, GC–MS analysis shows that the petroleum ether extract of *C. calcitrapa* is primarily composed of fatty acids and their derivatives, along with trace amounts of hydrocarbons, terpenoids, and aromatic compounds, which may collectively contribute to its reported biological activities.

**Table 3:** Chemical constituents identified by GC–MS in the petroleum ether extract of *C. calcitrapa.*

No	RT	Compound name	Area%	Molecular weight (g/mol)	Molecular formula
1	4.929	Decane	0.356	142.28	C_10_H_22_
2	6.735	Dodecane	0.151	170.33	C_12_H_26_
3	7.085	Naphthalene, decahydro-	0.158	138.25	C_10_H_18_
4	8.841	Undecane	0.516	156.31	C_11_H_24_
5	9.496	1-Methyldecahydronaphthalene	0.123	152.28	C_11_H_20_
6	12.417	Tridecane	0.521	184.36	C_13_H_28_
7	12.777	Undecane, 2,6-dimethyl-	0.127	184.36	C_13_H_28_
8	14.398	Pentadecane	0.184	212.41	C_15_H_32_
9	19.255	Octadecane	0.291	254.5	C_18_H_38_
10	20.175	Benzene, (1-butylhexyl)-	0.495	218.38	C_16_H_26_
11	20.365	Benzene, (1-propylheptyl)-	0.331	218.38	C_16_H_26_
12	20.740	Benzene, (1-propyloctyl)-	0.306	232.4	C_17_H_28_
13	20.921	Dodecanoic acid	0.189	200.32	C_12_H_24_O_2_
14	21.446	Benzene, (1-methylnonyl)-	0.485	246.4	C_18_H_30_
15	21.946	Benzene, (1-pentylheptyl)-	0.486	246.4	C_18_H_30_
16	22.011	Benzene, (1-butylheptyl)-	1.098	232.4	C_17_H_28_
17	22.196	Benzene, (1-heptyloctyl)-	0.926	288.5	C_21_H_36_
18	22.581	Benzene, (1-ethyldecyl)	0.944	326.5	C_18_H_30_O_3_S
19	23.146	Heneicosane	0.226	296.6	C_21_H_44_
20	23.236	Benzene, (1-methyldecyl)-	0.792	232.4	C_17_H_28_
21	23.622	Benzene, (1-hexylheptyl)-	1.013	260.5	C_19_H_32_
22	23.702	Benzene, (1-butyloctyl)-	0.919	246.4	C_18_H_30_
23	24.641	Benzene, (1-octyldodecyl)-	0.770	358.6	C_26_H_46_
24	24.277	Benzene, (1-ethyldecyl)-	0.587	246.4	C_18_H_30_
25	24.537	Tetradecanoic acid	3.149	228.37	C_14_H_28_O_2_
26	24.922	Benzene, (1-methylundecyl)-	0.675	246.4	C_18_H_30_
27	25.212	Benzene, (1-pentyloctyl)-	0.817	260.5	C_19_H_32_
28	25.317	Benzene, (1-butylnonyl)-	0.452	260.5	C_19_H_32_
29	25.397	3,7,11,15-Tetramethyl-2-hexadecen-1-ol	0.374	296.5	C_20_H_40_O
30	25.537	Benzene, (1-propylheptadecyl)-	0.561	358.6	C_26_H_46_
31	25.813	Cyclopropanenonanoic acid,2-[(2-butylcyclopropyl)methyl]-, methyl ester	0.142	322.5	C_21_H_38_O_2_
32	25.938	Benzene, (1-ethylundecyl)-	0.302	260.5	C_19_H_32_
33	26.128	Pentadecanoic acid	0.294	242.4	C_15_H_30_O_2_
34	26.258	1,6-Dioxaspiro[4.4]non-3-ene, 2-(2,4- hexadiynylidene)-	0.138	200.23	C_13_H_12_O_2_
35	26.693	Benzene, (1-methyldodecyl)-	0.789	260.5	C_19_H_32_
36	27.843	Hexadecenoic acid, *Z*-11-	0.183	254.41	C_16_H_30_O_2_
37	28.904	n-Hexadecanoic acid	15.834	256.42	C_16_H_32_O_2_
38	29.544	Hexadecane,2,6,10,14-tetramethyl-	0.193	282.5	C_20_H_42_
39	30.735	9,12-Octadecadienoic acid (*Z,Z*)-, methyl ester	0.129	294.5	C_19_H_34_O_2_
40	30.940	Heptadecanoic acid	0.204	270.5	C_17_H_34_O_2_
41	31.280	Phytol	0.561	296.5	C_20_H_40_O
42	32.390	9,12,15-Octadecatrienoic acid, 2,3-dihydroxypropyl ester, (*Z,Z,Z*)-	0.169	352.5	C_21_H_36_O_4_
43	34.781	Oleic Acid	48.426	282.5	C_18_H_34_O_2_
44	35.576	4 *α*,7,7,10 *α*-Tetramethyldodecahydrobenzo[*f*]chromen-3-ol	2.041	266.4	C_17_H_30_O_2_
45	36.342	Tricosane	1.680	324.6	C_23_H_48_
46	36.912	Octadecane, 3-ethyl-5-(2-ethylbutyl)-	0.922	366.7	C_26_H_54_
47	37.727	12-Methyl-*E,E*-2,13-octadecadien-1-ol	0.918	280.5	C_19_H_36_O
48	38.433	Geranyl isovalerate	0.448	238.37	C_15_H_26_O_2_
49	39.268	2,2,6-Trimethyl-1-(3-methylbuta-1,3-dienyl)-7- oxabicyclo[4.1.0]heptan-3-ol	0.174	222.32	C_14_H_22_O_2_
50	40.904	Dasycarpidan-1-methanol, acetate (ester)	0.153	326.4	C_20_H_26_N_2_O_2_

Gas chromatography–mass spectrometry (GC–MS) represents a robust analytical tool for the separation, identification, and characterization of volatile and semi-volatile phytochemicals in plant extracts. This technique has been widely used in phytochemical studies to explore hydrocarbons, fatty acids, alcohols, esters, and terpenoid compounds, which are known for their essential roles in plant metabolism and the biological activities of medicinal plants^87,88^. Analysis of the petroleum ether extract of *C. calcitrapa* using GC–MS revealed the presence of predominantly lipophilic components, particularly oleic acid (48.43%) and hexadecanoic (palmitic) acid (15.83%). These long-chain fatty acids, especially oleic acid, are known for their anti-inflammatory and antioxidant properties, which may contribute to the extract’s remarkable biological activity^14^. Previous phytochemical studies of *C. calcitrapa* and related species have highlighted an abundance of phenolic compounds and flavonoids, which are closely associated with in vitro antioxidant capacity. In particular, methanolic and other polar extracts of *C. calcitrapa* have demonstrated high total phenolic and flavonoid content, along with significant DPPH free radical scavenging activity comparable to standard antioxidants^14^. This supports our findings that even nonpolar extracts contain bioactive lipophilic compounds that may complement the antioxidant capacity attributed to polar phenolic compounds.

Besides fatty acids, phytol and other terpenoid derivatives detected in the petroleum ether extract are consistent with previous reports on *C. calcitrapa*, where terpenoids and related bioactive constituents have been associated with antioxidant, antimicrobial, and cytotoxic activities. GC–MS analyses of ethanolic extracts also revealed polyphenolic and other compounds contributing to antioxidant effects and modulation of oxidative stress related cellular processes^89^.

These results are consistent with a growing body of published studies on *Centaurea* species, where a variety of plant components contribute to antioxidant, antimicrobial, and possibly pharmacological effects, warranting further research into their therapeutic applications^90^.

### HPLC analysis of *C. calcitrapa* methanolic extract

**Fig. 4:**
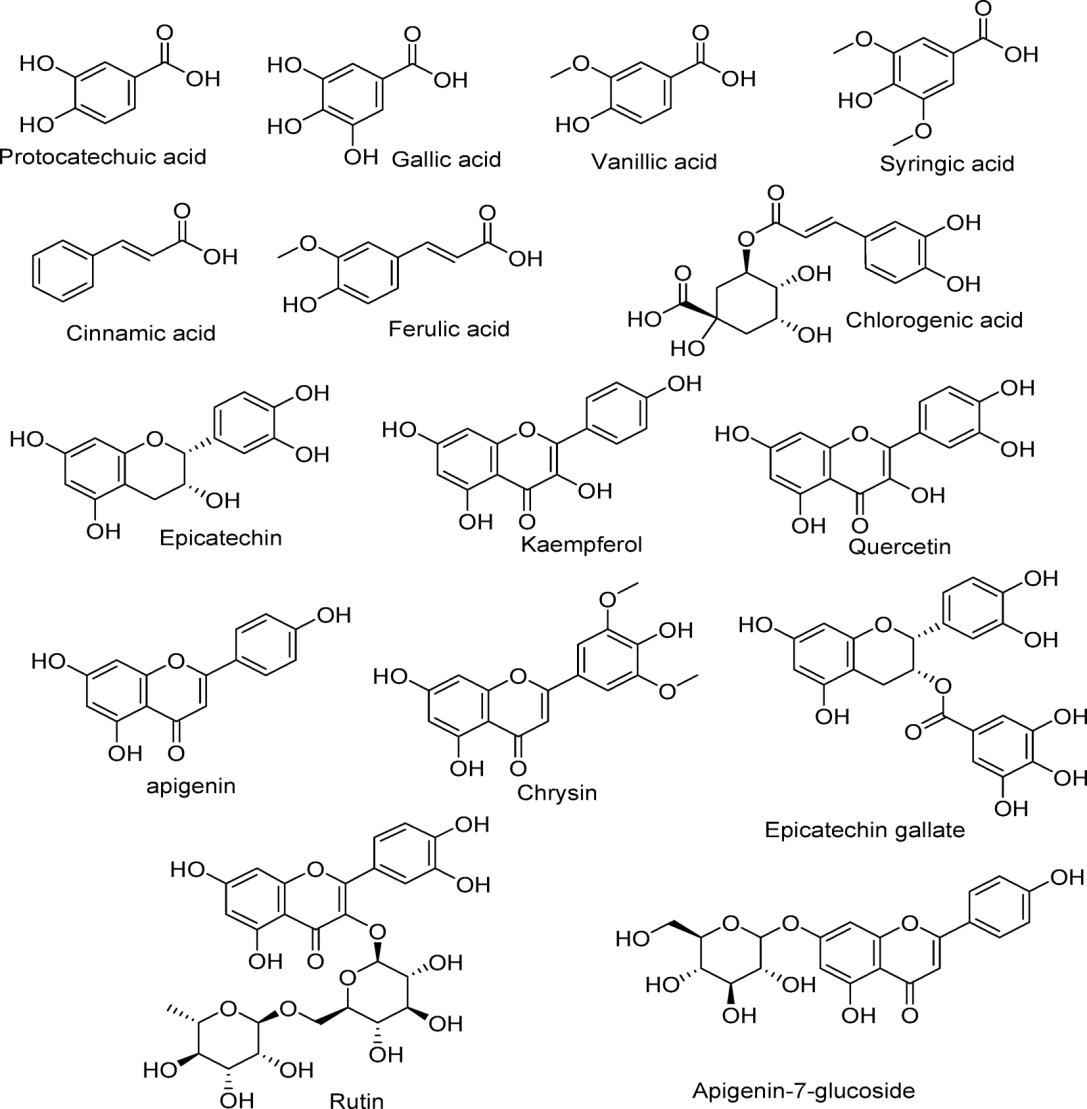
Chemical structures of phenolic constituents identified by HPLC in the methanolic extract obtained from the flowering aerial parts of *C. calcitrapa.*

HPLC analysis of the methanolic extract of *C. calcitrapa* aerial flowering parts revealed a variety of phenolic compounds. Table 4 show the identification and concentration of fifteen phenolic compounds present in the methanolic extract namely: flavonoids such as rutin (1222.05 µg/g), apigenin (843.48 µg/g), epicatechin (31.47 µg/g), epicatechin gallate (310.12 µg/g), apigenin-7-glucoside (180.36 µg/g), kaempferol (261.77 µg/g), quercetin (267.05 µg/g), and chrysin (10.37 µg/g), phenolic acid and its derivatives such as gallic acid (16.83 µg/g), chlorogenic acid (25.46 µg/g), syringic acid (8.87 µg/g), ferulic acid (6.71 µg/g), protocatechuic (308.02 µg/g), cinnamic acid (4.22 µg/g), and vanillic acid (26.48 µg/g). Figure 4 shows the chemical structures detected in the methanolic extract using HPLC.

**Table 4:** Phenolic profiling of the methanolic extract obtained from the flowering aerial parts of *C. calcitrapa.*

Compounds	Retention time (min)	Concentration (µg/g)
Gallic acid	3.94	16.83
Protocatechuic	7.06	308.02
Chlorogenic acid	14.08	25.46
Syringic acid	16.09	8.87
Epicatechin	16.71	31.47
Vanillic acid	17.43	26.48
Ferulic acid	22.27	6.71
Epicatechin gallate	24.19	310.12
Rutin	25.20	1222.05
Apigenin-7-glucoside	30.04	180.36
Cinnamic acid	35.59	4.22
Qurecetin	36.69	267.05
Apigenin	40.87	843.48
Kaempferol	41.65	261.77
Chrysin	53.44	10.37

High-performance liquid chromatography (HPLC) is a well-established analytical approach for the qualitative and quantitative identification of phenolic compounds and flavonoids in medicinal plants. Phenolic and flavonoid compounds are known for their biologically active roles in medicinal plants, contributing to their antioxidant, antimicrobial, anti-inflammatory, and pharmacological properties^91,92^. Rutin is a flavonol glycoside that combines quercetin and sugars and has been widely documented for its antioxidant and anti-inflammatory properties. It significantly contributes to the reduction of oxidative stress and may support vascular health^93^. This pattern is consistent with previous studies on *Centaurea parviflora* Desf.^94^. Gallic acid (3,4,5-trihydroxybenzoic acid) is a naturally occurring polyphenol with potent antioxidant activity. Due to its multiple hydroxyl groups, it exhibits strong free radical scavenging, anti-inflammatory, anticancer, and antibacterial effects, and has been linked to protective roles in neurological and cardiovascular disorders^95^. Apigenin is a flavonoid with proven anti-inflammatory and anti-cancer properties. It has been shown to influence signaling pathways related to cell cycle regulation, making it an important indicator of effective plant extracts^96^. Kaempferol is a flavonol associated with antioxidant, anti-atherosclerotic, and anticancer properties. It helps prevent the oxidation of low-density lipoprotein (LDL), thus contributing to cardiovascular protection^97^. This is similar to previous findings, where phenolic profiling of *Centaurea glastifolia* revealed the presence of kaempferol-3-O-glucoside, confirming that kaempferol derivatives occur in the genus and likely contribute to its biological activities^98^. Syringic acid is an antioxidant that may help protect against cancer cell growth and cell damage; although it is usually found in low concentrations, it enhances the overall phenolic activity of plant extracts^99^.

### Antioxidant activity of *C. calcitrapa* methanolic extract

**Fig. 5:**
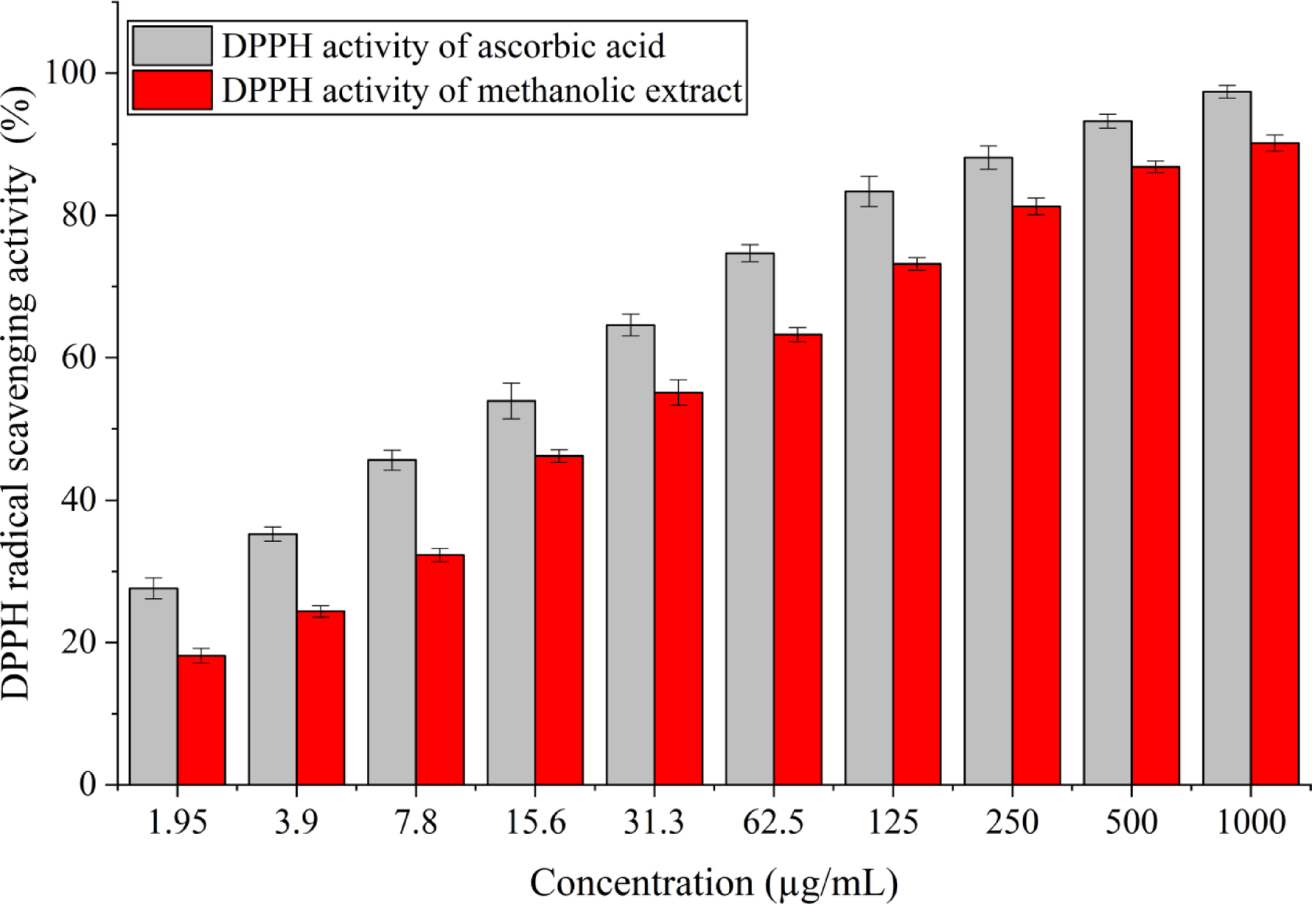
DPPH radical scavenging activity of the methanolic extract of *C. calcitrapa* at various concentrations compared with ascorbic acid. Data are expressed as mean ± standard deviation (n = 3).

In the present study, the DPPH radical scavenging activity of the methanolic extract was evaluated and compared with ascorbic acid. The IC_50_ values, representing the concentration required to inhibit 50% of DPPH radicals, were determined over a concentration range of 1.95–1000 µg/mL, and the results are presented in Fig. 5. The DPPH scavenging activity of methanolic extract of at concentrations of 62.5, 125, 250, 500, and 1000 µg/mL were 63.25 ± 1.0%, 73.17 ± 0.88%, 81.27 ± 1.17%, 86.82 ± 0.83%, and 90.16 ± 1.13%, respectively. The IC_50_ of ascorbic acid was 12.71 ± 0.7 µg/mL comparison to MeOH extract was 24.01 ± 0.73 µg/mL.

**Fig. 6:**
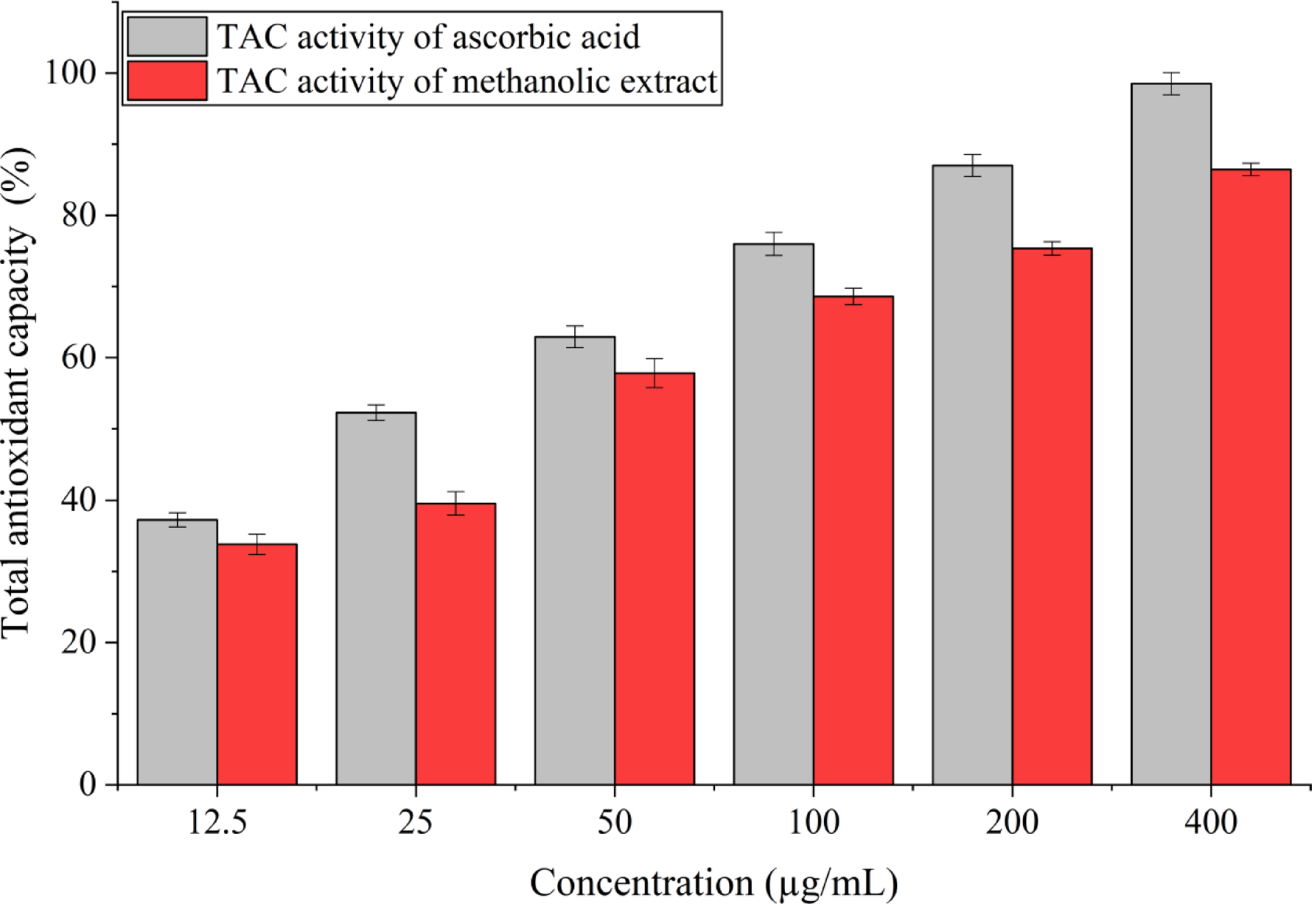
Total antioxidant capacity of the methanolic extract of *C. calcitrapa* at various concentrations compared with ascorbic acid. Data are expressed as mean ± standard deviation (n = 3).

As shown in Fig. 6, the total antioxidant capacity (TAC) of the methanolic extract of *C. calcitrapa* was evaluated in comparison with ascorbic acid as a standard. The IC_50_ values, representing the concentration required to achieve 50% inhibition, were determined over a concentration range of 12.5–400 µg/mL. The TAC of the methanolic extract at a concentration of 400 µg/mL was 86.45 ± 0.88%, compared with 98.51 ± 1.56% for ascorbic acid. The IC_50_ value was 37.58 ± 1.21 µg/mL, compared with ascorbic acid (23.29 ± 0.72 µg/mL).

The antioxidant activity of the methanolic extract of *C. calcitrapa* was estimated using both DPPH free radical scavenging activity and TAC assays and compared to ascorbic acid as a standard antioxidant in order to obtain a comprehensive overview of its antioxidant behavior. The DPPH test demonstrates a strong free radical scavenging capacity, reflecting the extract’s ability to donate hydrogen atoms or electrons to neutralize free radicals. This test is one of the most common and reliable methods for evaluating the antioxidant activity of phenolic-rich extracts, as phenolic acids and flavonoids are known to act as key free radical scavengers^100,101^. At the same time, the TAC test showed a high overall reduction capacity, indicating that the antioxidant effect of the extract is not limited to a single pathway. The TAC index reflects the cumulative activity of all active compounds in the redox reactions present, including phenolics, flavonoids and other secondary metabolites^102^. In this study, the methanolic extract of *C. calcitrapa* demonstrated a remarkable ability to inhibit DPPH free radicals by more than 85% in a free radical inhibition assay. Similar findings were previously published by Mekky et al.^14^. Furthermore, as shown in Fig. 6, the TAC assay confirmed that the methanolic extract of *C. calcitrapa* exhibited significant antioxidant activity. These results are consistent with previous reports on *Centaurea aksoyi* and *Centaurea amaena*, which also demonstrated remarkable antioxidant capacity^103^.

### Antibacterial activity of *C. calcitrapa* methanolic extract

**Fig. 7:**
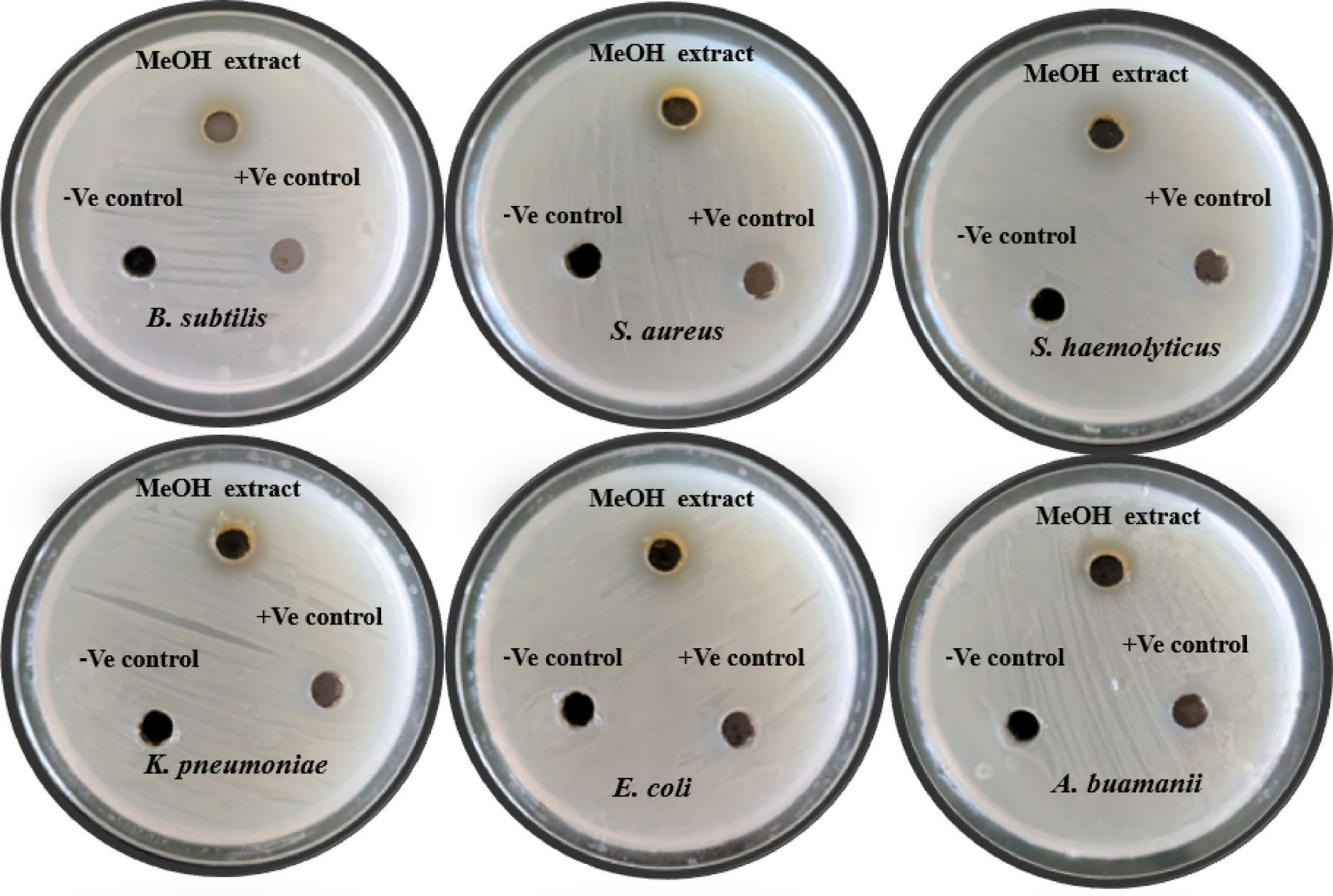
Inhibitory zones generated by the *C. calcitrapa* methanolic extract against the tested pathogenic bacteria.

The antibacterial properties of six human pathogenic bacteria were evaluated in vitro using a methanolic extract of *C. calcitrapa*. Table 5 and Fig. 7 showed that the MeOH extract of *C. calcitrapa* had moderate antibacterial potential against both Gram-positive and Gram-negative bacteria, with an inhibition zone ranging from 16.89 ± 0.95 to 20.98 ± 1.52 mm.

**Table 5:** Antibacterial activity of the methanolic extract of *C. calcitrapa* against the tested pathogenic bacteria.

No	Isolate name	Diameter of Inhibition zone (mm) by MeOH crude extract (100 µL)		
		MeOH extract	+ ve Control (Ciprofloxacin)	-ve Control (DMSO)
1	*B. subtilis* (ATCC 6633)	17.62 ± 0.74^abcd^	15.27 ± 1.04^bcde^	0
2	*S. aureus* (ATCC25923)	17.58 ± 1.23^abcd^	17.98 ± 1.52^abc^	0
3	*S. haemolyticus* (ATCC29970)	20.98 ± 1.52^a^	13.37 ± 1.18^e^	0
4	*K. pneumoniae* (ATCC2146)	18.2 ± 1.27^ab^	14.51 ± 0.42^de^	0
5	*E. coli* (ATCC8739)	17.34 ± 1.22^bcd^	14.49 ± 1.39^de^	0
6	*A. baumannii* (ATCC 19,606)	16.89 ± 0.95^bcd^	14.57 ± 1.14^cde^	0

Values are presented as mean ± SD (n = 3). Different superscript letters (a–e) within the same column indicate significant differences (P 0.05).

Table 6 illustrated that the MIC values ranged from 62.5 ± 1.0 to 125 ± 3.61 mg/mL. The lowest MIC (62.5 mg/mL) was recorded against *S. aureus*, *S. haemolyticus*, *E. coli*, and *A. baumannii*, indicating higher sensitivity of these strains to the extract. In contrast, *B. subtilis* and *K. pneumoniae* showed higher MIC values (125 mg/mL), suggesting comparatively lower susceptibility. Similarly, the MBC values ranged from 125 ± 2.0 to 250 ± 3.61 mg/mL. The lowest MBC (125 mg/mL) was observed for *S. aureus*, *S. haemolyticus*, *E. coli*, and *A. baumannii*, whereas *B. subtilis* and *K. pneumoniae* required higher concentrations (250 mg/mL) for bactericidal activity.

**Table 6:** Minimum inhibitory concentrations (MICs) and minimum bactericidal concentrations (MBCs) of the methanolic extract from *C. calcitrapa* against selected human pathogenic bacterial strains.

No	Isolate name	MIC and MBC of MeOH crude extract (mg/mL)	
		MIC	MBC
1	*B. subtilis* (ATCC 6633)	125	250
2	*S. aureus* (ATCC25923)	62.5	125
3	*S. haemolyticus* (ATCC29970)	62.5	125
4	*K. pneumoniae* (ATCC2146)	125	250
5	*E. coli* (ATCC8739)	62.5	125
6	*A. baumannii* (ATCC 19,606)	62.5	125

MIC and MBC values are expressed as endpoint concentrations obtained from two independent experiments using broth microdilution assay.

Examining the antibacterial properties of plant extracts is crucial in phytochemical and biological research, as medicinal plants are potential sources of novel antimicrobial agents. This importance has been amplified by the global rise in antibiotic-resistant bacteria, creating a pressing need for alternative and complementary natural products^104^. In this experiment, the methanolic extract of *C. calcitrapa* showed moderate antibacterial activity against the tested pathogenic bacteria, with the zone of inhibition ranging from 16.89 ± 0.95 to 20.98 ± 1.52 mm, and the lower MIC and MBC values indicate antibacterial potency^105,106^. Similarly, Mekky et al.^14^ found that *C. calcitrapa* extracts are effective against both Gram-negative and Gram-positive bacteria. Furthermore, lower MIC and MBC values of the methanolic extract against *S. aureus*, *S. haemolyticus*, *E. coli*, and *A. baumannii* indicate higher susceptibility of these strains compared with *B. subtilis* and *K. pneumoniae*. The antibacterial activity may be attributed to phenolic compounds, which disrupt bacterial cell walls and membranes, inhibit enzyme activity, and induce oxidative stress in microbial cells^107,108^

### Antidiabetic activity of *C. calcitrapa* methanolic extract

**Fig. 8:**
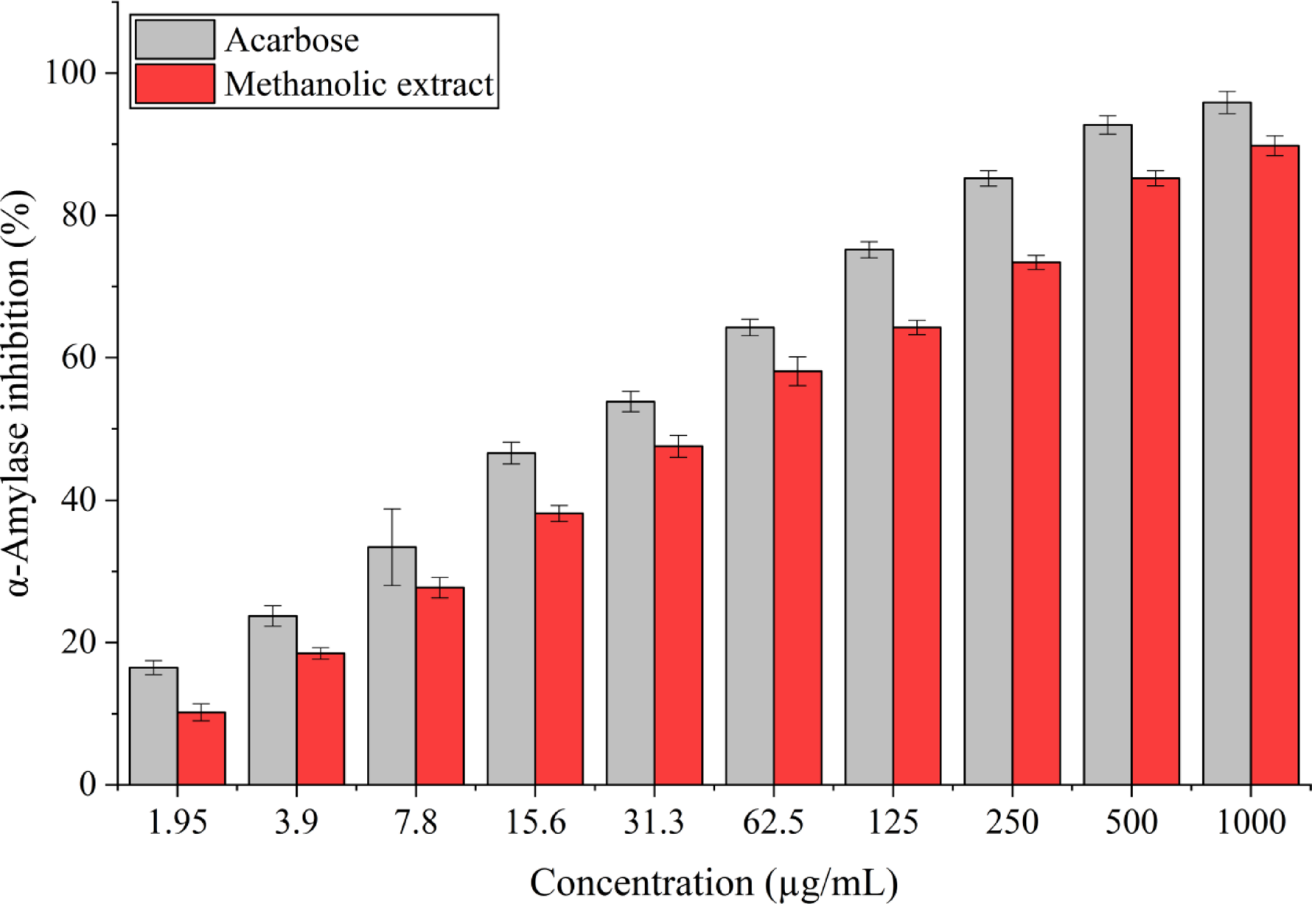
α-Amylase inhibitory activity of *C. calcitrapa* methanolic extract and Acarbose at 1.95 to 1000 µg/mL. Data are expressed as mean ± standard deviation (n = 3).

The inhibition of α-amylase by methanolic extract of *C. calcitrapa* was measured compared to Acarbose at concentrations ranging from 1.95 to 1000 µg/mL, as shown in Fig. 8. The α-amylase inhibition activity of methanolic extract of *C. calcitrapa* at concentrations of 250, 500, and 1000 µg/mL was 73.38 ± 0.98, 85.20 ± 1.07, and 89.79 ± 1.39, respectively. The IC_50_ value for Acarbose was 24.08 ± 1.01 µg/mL, while it was 37.26 ± 0.91 µg/mL for the methanol extract.

**Fig. 9:**
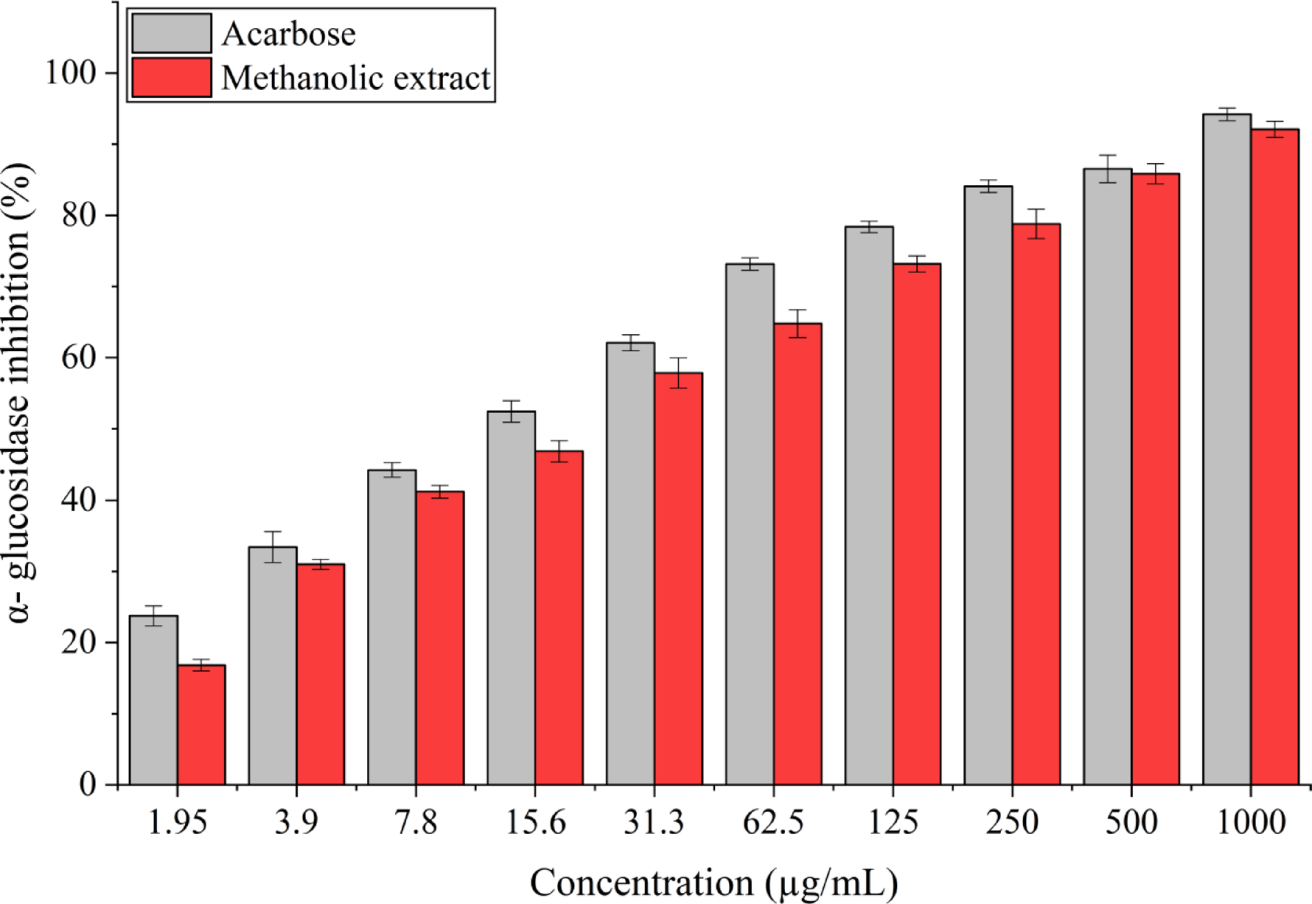
α-glucosidases inhibitory activity of *C. calcitrapa* methanolic extract and Acarbose at 1.95 to 1000 µg/mL. Data are expressed as mean ± standard deviation (n = 3).

Figure 9 illustrates the inhibitory effect of the methanolic extract of *C. calcitrapa* on α-glucosidase. The extract exhibited inhibition rates of 78.8 ± 2.08%, 85.84 ± 1.43%, and 92.09 ± 1.12% at concentrations of 250, 500, and 1000 µg/mL, respectively. The IC_50_ value for the methanolic extract was 20.19 ± 1.54 µg/mL, compared to 13.49 ± 0.71 µg/mL for Acarbose.

The methanolic extract showed concentration-dependent in vitro inhibitory activity against α-amylase, reaching 89.79 ± 1.39% inhibition at 1000 µg/mL, with an IC_50_ value (37.26 ± 0.91 µg/mL) relatively close to that of the standard drug Acarbose (24.08 ± 1.01 µg/mL). Similarly, strong inhibition of the α-glucosidase enzyme was observed, with inhibition reaching 92.09% at a concentration of 1000 µg/mL, and the IC_50_ value (20.19 ± 1.54 µg/mL) approaching that of Acarbose (13.49 ± 0.71 µg/mL). It is noteworthy that the extract showed relatively stronger inhibition of α-glucosidase compared to α-amylase, which may be considered advantageous, as selective α-glucosidase inhibition has been associated in the literature with reduced gastrointestinal side effects commonly observed with strong α-amylase inhibition^109,110^.

The observed in vitro carbohydrate-hydrolyzing enzyme inhibitory activity of *C. calcitrapa* may be attributed to its rich phytochemical composition, particularly the phenolic acids, flavonoids, and sesquiterpene lactones found in species of the genus *Centaurea*. Polyphenolic compounds are known to bind to digestive enzymes via hydrogen bonds and hydrophobic interactions, thereby reducing carbohydrate breakdown and glucose absorption^111,112^. In addition, flavonoid compounds such as quercetin and kaempferol derivatives have shown significant inhibitory effects against both α-amylase and α-glucosidase enzymes^113,114^ Previous studies on *Centaurea* species have indicated antioxidant and blood glucose-lowering properties, supporting the current findings. The dual antioxidant and enzyme-inhibiting properties are particularly significant, as oxidative stress plays a pivotal role in the development of diabetes and its complications^66^.

## Conclusion

This study demonstrates that the flowering aerial parts of *C. calcitrapa* possess high nutritional value and a rich phytochemical composition. Preliminary analysis revealed significant amounts of fiber, protein, carbohydrates, and essential minerals, suggesting potential relevance for nutritional and functional research applications. Qualitative and quantitative phytochemical analyses confirmed the abundance of phenolic compounds, flavonoids, flavanols, and tannins, which are widely recognized for their biological activities. Chromatographic analysis confirmed these findings, with GC–MS identifying lipophilic compounds such as oleic and palmitic acids, while HPLC detected phenolic constituents including rutin, apigenin, quercetin, and protocatechuic acid. The methanolic extract showed strong antioxidant activity, significant antibacterial effects against both Gram-positive and Gram-negative bacteria, in addition to in vitro carbohydrate-hydrolyzing enzyme inhibitory activity against α-amylase and α-glucosidase. Overall, these findings indicate that *C. calcitrapa* is a promising source of bioactive constituents. However, the present study is limited to chemical characterization and in vitro assays; therefore, the results should be interpreted as preliminary biochemical evidence rather than confirmation of therapeutic efficacy. Further studies, including isolation and characterization of active compounds, mechanistic investigations, cytotoxicity evaluation, and in vivo studies, are required to validate these biological activities and explore their potential applications in food and pharmaceutical research.

## Data Availability

The data supporting the findings of this study are available from the corresponding author upon reasonable request.
